# The role of *TP53* gain-of-function mutation in multifocal glioblastoma

**DOI:** 10.1007/s11060-019-03318-5

**Published:** 2020-01-30

**Authors:** Lauren R. Olafson, Manuri Gunawardena, Sheri Nixdorf, Kerrie L. McDonald, Robert W. Rapkins

**Affiliations:** Cure Brain Cancer Biomarkers and Translational Research Group, Prince of Wales Clinical School, University of New South Wales, Sydney, NSW 2052 Australia

**Keywords:** Glioblastoma, Multifocal, TP53, Gain-of-function

## Abstract

**Purpose:**

The phenotypic and genotypic landscapes in multifocal glioblastoma (MF GBM) cases can vary greatly among lesions. In a MF GBM patient, the rapid development of a secondary lesion was investigated to determine if a unique genetic signature could account for the apparent increased malignancy of this lesion.

**Methods:**

The primary (G52) and secondary (G53) tumours were resected to develop patient derived models followed by functional assays and multiplatform molecular profiling.

**Results:**

Molecular profiling revealed G52 was wild-type for *TP53* while G53 presented with a *TP53* missense mutation. Functional studies demonstrated increased proliferation, migration, invasion and colony formation in G53.

**Conclusion:**

This data suggests that the *TP53* mutation led to gain-of-function phenotypes and resulted in greater overall oncogenic potential of G53.

**Electronic supplementary material:**

The online version of this article (doi:10.1007/s11060-019-03318-5) contains supplementary material, which is available to authorized users.

## Introduction

Glioblastoma (GBM) is the most common and malignant primary brain tumour harbouring few effective treatment options and a poor prognosis. Although often presenting as a solitary tumour (sGBM), GBM can exist as multiple lesions (mGBM), further decreasing the dismal 15 month prognosis to a mere 6–8 months [1–3]. mGBMs are more likely to be deeply disseminated in vital structures, thus preventing gross total resection and resulting in a poorer prognosis compared to sGBM [2]. Incidence of mGBM in literature ranges from 0.5 to 20% with a more recent study reporting incidence as high as 35% [3–5]. Though this apparent increase in incidence likely results from technological advancements in neuroimaging, this prevalence warrants further genetic investigation into mGBM [2].

Dating back to 1963, Batzdorf and Malamud proposed classification of multiple gliomas into multifocal (MF) and multicentric (MC) based on pathological criteria [6]. Maintaining this criterion to present day, MF tumours display a pattern of dissemination whereas MC tumours display no continuity between lesions in the context of time or space, both of which are identified by T2/FLAIR-weighted signals on magnetic resonance imaging (MRI) [6]. With advancements in next generation sequencing (NGS), studies suggest that genetically distinct mGBMs are rare with most cases likely being MF as opposed to truly MC [2, 7]. In the present case, two lesions lacked anatomical continuity in imaging diagnostics which, by clinical definition, are considered MC. However, multiplatform molecular examination revealed nearly identical genomic profiles, a case similarly reported by Akimoto and colleagues [7]. These findings suggest that the secondary tumour may have developed either from a subpopulation of the primary tumour or from the evolution of a migrated tumour precursor cell. Considering the genetic similarities between these tumours, this case will be referred to as MF for the purpose of this study. Nevertheless, the definitions of MC or MF offer very little clinical value.

The genomic profiles of GBM have a vast spectrum resulting from significant heterogeneity within the inter- and intra-tumoural landscape. Additionally, the diffusely infiltrative nature of the disease has had significant implications on diagnostic and therapeutic advancements. Despite improved surgical techniques and anticancer drugs, current treatment options for sGBM are limited to resection followed by concurrent radiotherapy and chemotherapy [1]. Treatment options for mGBM are inevitably more complicated and remain controversial [5]. Where the 5-year survival rate has improved for other malignancies including breast and prostate cancer, no improvements for GBM have been recorded in the past 20 years. Thus, an understanding of the aggressive biology and tumour evolution of this formidable disease is urgently needed.

Coined the “guardian of the genome”, the tumour suppressor gene *TP53* has garnered significant research attention. It is the most commonly mutated gene found in all human cancers and consequently, the most extensively studied gene of all time. Alterations in *TP53* and its encoding protein, p53, have been found in approximately half of all human cancers with the majority of mutant proteins resulting from missense mutations [8–10]. Previous studies have demonstrated that these alterations not only result in a loss of wild-type (wt) function, but can acquire gain of function (GOF) phenotypes rendering the cancer more aggressive [11]. These GOF phenotypes may potentiate aggressive tumour progression through increased cell migration, proliferation, invasion and metastasis providing the mutant isoforms with greater oncogenic potential than p53 wt loss [11–14].

Most of the investigative findings on p53 GOF phenotypes have been conducted through in vitro and in vivo studies, but little has been reported within a more relevant clinical setting. Herein, we present a 55-year-old male patient with MF GBM presenting in the left thalamic (4.7 cm) and left temporal (5.4 cm) regions with the latter developing in less than two weeks after discovery of the thalamic lesion. Following resection, fresh primary and secondary tumour samples were collected and processed for patient derived model development. Multi-platform molecular profiling was conducted on both samples with additional standard of care diagnostics. Molecular profiling revealed a *TP53* missense mutation with subsequent functional studies identifying p53 GOF phenotypes in the secondary tumour. This case report emphasises the complex GBM landscape and thus, the potential contribution of genetic analysis and interpretation in formulating personalised treatment plans.

## Materials and methods

### Patient-derived cell line establishment and tissue culture

**Fig. 1:**
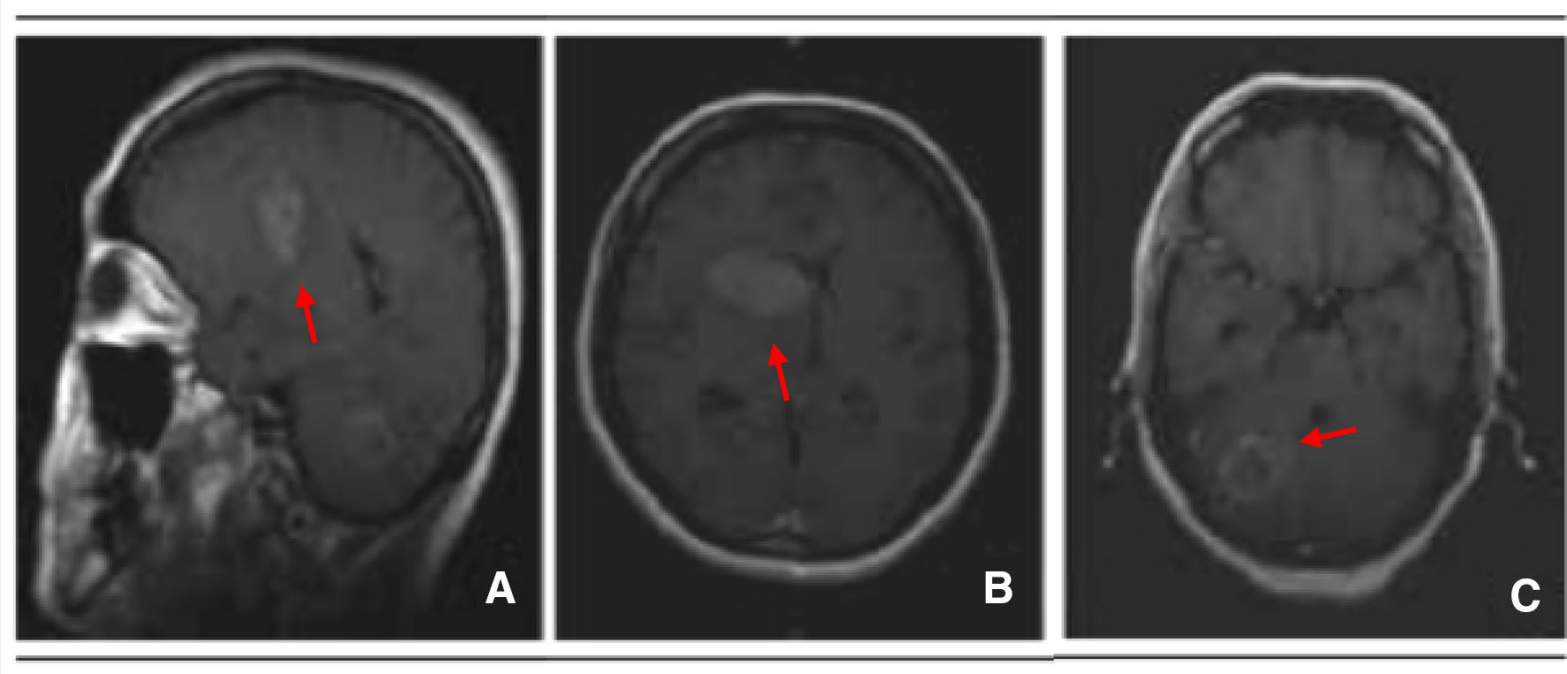
Pre-operative MRI scans exhibiting two expansive bulky lesions arising in the left thalamus (**a** and **b**) and cerebella hemisphere **c** with an irregular ring contrast enhancement

A 55-year-old male patient presented with left-sided headache, dysphasia, significant right proximal arm weakness and peri-orbital pain. Imaging revealed an enlarged left thalamic lesion 4.7 cm in diameter. The patient was scheduled for surgical resection within two weeks at The Prince of Wales Private Hospital (Randwick, Australia). The Human Research Ethics Committee, South Eastern Sydney Local Health District–Northern Sector approved the collection and use of fresh human GBM tissue for this project (HREC No: 2008-094). Pathology confirmed extensive palisading necrosis and vascular proliferation consistent with GBM, World Health Organisation grade IV. Preoperative imaging on the day of surgery revealed an additional lesion in the left temporal region measuring 5.4 cm in diameter (Fig. 1). Samples 2–3 cm in diameter from both lesions were collected and transported on ice for processing within 30–60 min of resection. Tissue fragments were washed with phosphate buffered saline (PBS) following the removal of necrotic and vascular regions under a dissecting microscope. A cell pellet was established and resuspended into 10 ml of serum-free media supplemented with 50 μl of epidermal growth factor and fibroblast growth factor before being plated onto a T75 flask pre-coated with Corning® Matrigel®. Low passage, patient-derived primary cell lines (PDCLs) were established as G52 (left thalamic) and G53 (left temporal) and maintained for subsequent analyses.

### Multiplatform molecular profiling of tumour

Formalin-fixed paraffin-embedded (FFPE) tumour tissue samples of G52 and G53 were sent for multiplatform profiling to Caris Life Sciences, Phoenix, AZ (Molecular Intelligence Service™). Commercially available antibodies and detection kits were used for immunohistochemical (IHC) analysis of PD-1 (NAT1 antibody, Cell Marque), PD-L1 (SP142, Spring Bioscience) and EGFR (Invitrogen) expression. PD-1 expression on the plasma membrane of tumour infiltrating lymphocytes was examined and the density recorded. PD-L1 membrane expression on > 5% of tumour cells was measured as positive [15]. EGFR expression was assessed using a H-score grading system between 0 and 300. To ensure validity of results, all IHC assays consisted of positive and negative controls. EGFR gene alterations were evaluated for copy number changes using in situ hybridisation assays: chromogenic (CISH [Ventana, Tucson, AZ]) and fluorescent (FISH [Abbott Molecular/Vysis]). Amplification of EGFR was recognised if > 10% of analysed cells contained > 15% EGFR gene copies per well or if the EGFR/CEP7 ratio was > 2 [16]. FISH was also performed to detect 1p19q co-deletion and both FISH/CISH were used to detect cMET gene amplification. EGFRvIII mutational analysis was performed on RNA extracted from tumour tissue samples using fragment analysis sequencing and multiplex ligation-dependent probe amplification. NGS analysis was performed on tumour DNA using the Illumina MiSeq platform. Specific regions of 594 genes were amplified (Supplementary Tables 1 and 2) using the Illumina TruSeq Amplicon-Cancer Hotspot Panel. A > 99% confidence was detected on all variants based on amplicon coverage and the frequency of mutations present. Sample regions sequenced achieved an average depth of coverage of > 1500x. Sanger sequencing was performed on selected regions of IDH2, KRAS, EGFR, c-KIT, BRAF, PIK3Ca and NRAS using PCR primers designed to amplify target sequences. Methylation testing of *MGMT* was achieved through pyrosequencing analysis of CpG sites. Samples were considered equivocal between ≥ 7% and < 9% methylation.

**Table 1:** Positive biomarkers and mutated genes analysed by pyrosequencing, in situ hybridisation, IHC and NGS for G52 and G53 tumour samples

Pyrosequencing	G52 Tumour	G53 Tumour
Pyro SEQ-*MGMT*	Unmethylated	Unmethylated
In situ hybridisation		
EGFR	Positive	Positive
IHC		
EGFR	Positive	Positive
PTEN	Positive	Positive
TOPIIA	Positive	Positive
PGP	Positive	Positive
Next generation sequencing		
*PTEN*	Mutated	Mutated
*EGFR*	Mutated	Mutated
*MAP3KI*	Mutated	Mutated
*NTRKI*	Mutated	Mutated
*TP53*	Wild-type	Mutated

### Amplification and sanger sequencing to validate TP53 mutations

Mutations in the *TP53* tumour suppressor gene were validated using isolated genomic DNA from G52 and G53 cell lines by PCR amplification followed by Sanger DNA sequencing. The most informative coding regions and intron/exon junctions of *TP53* (exons 5 through to 8) were amplified. The most frequent sites for mutation were represented by three hot spots at amino acids 175 (exon 5), 248 (exon 7) and 273 (exon 8). The specifically designed primer pairs used were 5′-TGT TCA CTT GTG CCC TGA CT-3′ (forward) and 5′-TAA CCC CTC CTC CCA GAG A-3′ (reverse) covering a 0.550-kilobase region of exons 5 and 6, pairs 5′-AGG CGC ACT GGC CTC ATC TT-3′ (forward) and 5′-TGT GCA GGG TGG CAA GTG GC-3′ (reverse) spanning a 0.283-kilobase region of exon 7 and pairs 5′- TTG GGA GTA GAT GGA GCC T-3′ (forward) and 5′-AGT GTT AGA CTG GAA ACT TT-3′ (reverse) covering a 0.445-kilobase region of exon 8. Real-time PCR amplification and detection was performed following the manufacturer’s protocol using KAPA-Taq Polymerase Kit (*Kapa* Biosystems): initial denaturation (2 min at 95 °C), 35 cycles of denaturation (95 °C), annealing (61 °C) and extension (72 °C) with a final extension cycle (1 min at 72 °C). The success of the PCR was verified by running a 5 μL aliquot of the PCR product on a 1.6% agarose gel. Sanger sequencing was performed on the PCR products following the manufacturer’s protocol using *BigDye*® Terminator Cycle Sequencing Kit v3.1 (Applied Biosystems), 45 cycles were performed using an annealing temperature of 56 °C. Mutational data was collected on ABI Prism 3730xl Genetic Analyzer (Applied Biosystems).

### Immunohistochemistry

IHC staining of FFPE sections for Ki-67, an astrocytic proliferative marker, was performed. The sections were deparaffinised with xylene then rehydrated with decreasing concentrations of alcohol. The slides were then washed for 5 min in PBS. Heat induced antigen retrieval was performed by immersing the sections in 10 ml of citrate buffer pH 6 (Dako, Glostrup, Denmark) and microwaving on high 2 × 5 min each. Slides were removed and allowed to cool in solution for 20 min then washed with PBS for 5 min. Endogenous peroxidase activity was exhausted by treating slides with 3% hydrogen peroxide solution. Tissues were then covered with two drops of endogenous biotin blocking agents, 0.1% Avidin and Biotin. After washing with PBS, the sections were blocked for 1 h using 2% Bovine Serum Albumin in PBS. Each slide was then incubated with the Ki-67 monoclonal primary antibody (clone MIB-1; DAKO, Glostrup Denmark) at room temperature for 1 h. Sections were then washed three times with PBS. A brown colour was developed by applying diaminobenzidine DAB chromogen (DAKO K3456) substrate for 5–10 min followed by haematoxylin counterstaining. As a result, Ki-67 positive nuclei appear brown. Ki-67 expression is defined as a percentage score of the total number of tumour cells with positive nuclear staining per 1000 cells. Viable infiltrating areas on the cancer tissue were selected using a × 10 objective magnification for analysis. Vascular components, haematogenous tumour cells and non-specific cytoplasm staining were excluded from the analysis. To determine the ratio of positive cells, × 40 magnification was used to count cells showing positive staining. From each chosen area, 1000 cells were counted in consecutive fields. Cells were considered Ki67 + if there was clearly detectable dark brown colouration of the nucleus.

### Cell proliferation assay

Cell Proliferation was assessed using the xCELLigence Real-Time Cell Analysis (RTCA) system (Roche, Switzerland) on Matrigel coated CIM-16 xCELLigence plates. The E-96 plate consists of incorporated gold cell sensor arrays which allow for the monitoring of cells inside each well. Electronic impedance of the sensors was measured through the detection of cells adhering to the electrodes. Cell attachment acts as insulation altering the electrode/solution interfaces, thereby increasing impedance. The E-96 plate was connected to the RTCA system and background impedance was measured. G52 and G53 cells were seeded onto the plate at an optimised density of 8 × 10^3^ cells per well. The plate was connected to the RTCA system and incubated at 37  °C. Cell adhesion, growth and proliferation were measured every 15 min for 48 h via the incorporated sensor electrode rays. Four replicates of the cell concentration were performed in each test. Electrical impedance was measured by the xCELLigence RTCA software as an arbitrary parameter labelled Cell Index (CI).

### Cell invasion and migration assay

Cell migration and invasion assays were conducted using RTCA as previously described. The experiment was performed on CIM-16 plates consisting of an upper and lower chamber separated by an artificial microporous membrane. Migration of cells was detected by microelectrodes attached to the underside of the membrane. Background signals generated by cell-free media were measured. For migration experiments, an optimised density of 4 × 10^4^ cells per well were seeded onto the upper chamber of the CIM-16 plates in serum free medium. The lower chambers were filled with 5% FCS, used as a chemoattractant. The invasion experiments followed an identical protocol with the additional application of a Matrigel layer to the upper side of the membrane. The chambers were incubated at 37 °C for a minimum of 4 h prior to seeding cells. Cells were seeded at an optimised density of 2 × 10^4^ cells per well. CI was measured over 35 h using RTCA software. Original datasets generated by xCELLigence were exported to MS Excel and reconstructed with data points corresponding to selected timepoints.

### Colony formation assay

The ability for a single cell to develop into a colony can be measured in vitro by colony formation assays. There must be at least 50 cells or more in a grouping to constitute a colony. Colony formation assays were performed on both G52 and G53 cell lines. Cells were harvested from culture at 80% confluency and trypsinised into single cell suspensions. A cell count was performed. Cells were seeded onto individual 6 well plates at concentrations of 50, 100, 200, 300, 400 and 600 cells/ml. Plates were incubated at 37 °C and cell attachment was analysed under a microscope after 24 h. Cells were left in a 37 °C incubator for 14 days. The plates were then stained with a 6% glutaraldehyde and 0.5% crystal violet mixture for 30 min and rinsed with water. A stereomicroscope and colony counting pen were used to count colonies.

## Results

### Biomarkers and mutations revealed by molecular profiling

A total of 27 biomarkers were tested in the samples using multiplatform molecular profiling involving in situ hybridisation, IHC, Sanger sequencing, fragment analysis and NGS with a further 594 genes amplified using the Illumina TruSeq Amplicon-Cancer Hotspot Panel. No *MGMT* promoter methylation was identified in either tumour and biomarkers that tested positive were identical in both (Table 1). Mutational analysis revealed 4 of the 594 genes analysed were mutated in both tumours. Further NGS analysis revealed that G52 somatic single nucleotide variations and indel mutations shared 92% similarity with G53. The only variance noted between the two tumours was observed in *TP53*, presenting as wt in G52 and mutated in G53. Altogether, the biomarker and mutational data revealed similar molecular profiles of the MF lesions, differing only in a *TP53* mutation.

### Mutational validation of TP53 in multifocal glioblastoma lesions

**Fig. 2:**
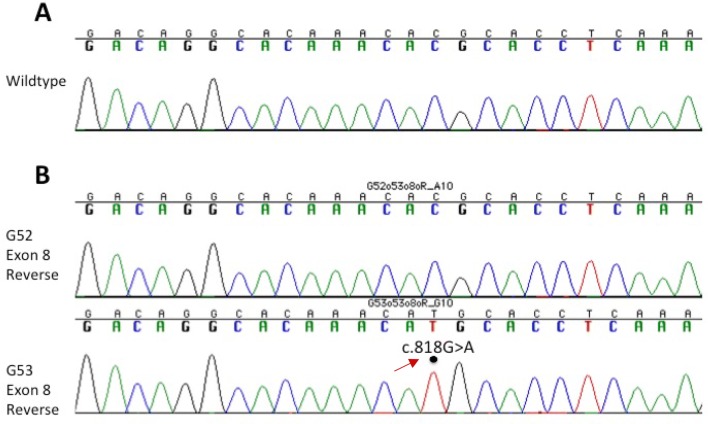
Nucleotide sequence analysis of *TP53* exon 8 in G52 and G53 tumour samples. **a** Wt nucleotide sequence at codon 273. **b** G52 and G53 tumour nucleotide sequence at codon 273. Dot indicates the position of the C to T nucleotide substitution and c.818G.A (p.R273H) mutation of the *TP53* gene in the G53 tumour

Genotyping of the ‘hotspot’ codons of *TP53* (175, 248 and 273) in both tumours confirmed missense mutation c.818G > A (p.R273H) in exon 8 of G53 (Fig. 2b). Homozygotic substitution of coding DNA sequence (C:G > T:A) has been previously annotated as a single nucleotide polymorphism (SNP) in the dbSNP database (rs28934576).

### Mutant p53 gene promotes tumour proliferation, migration and invasion in human GBM cells

**Fig. 3:**
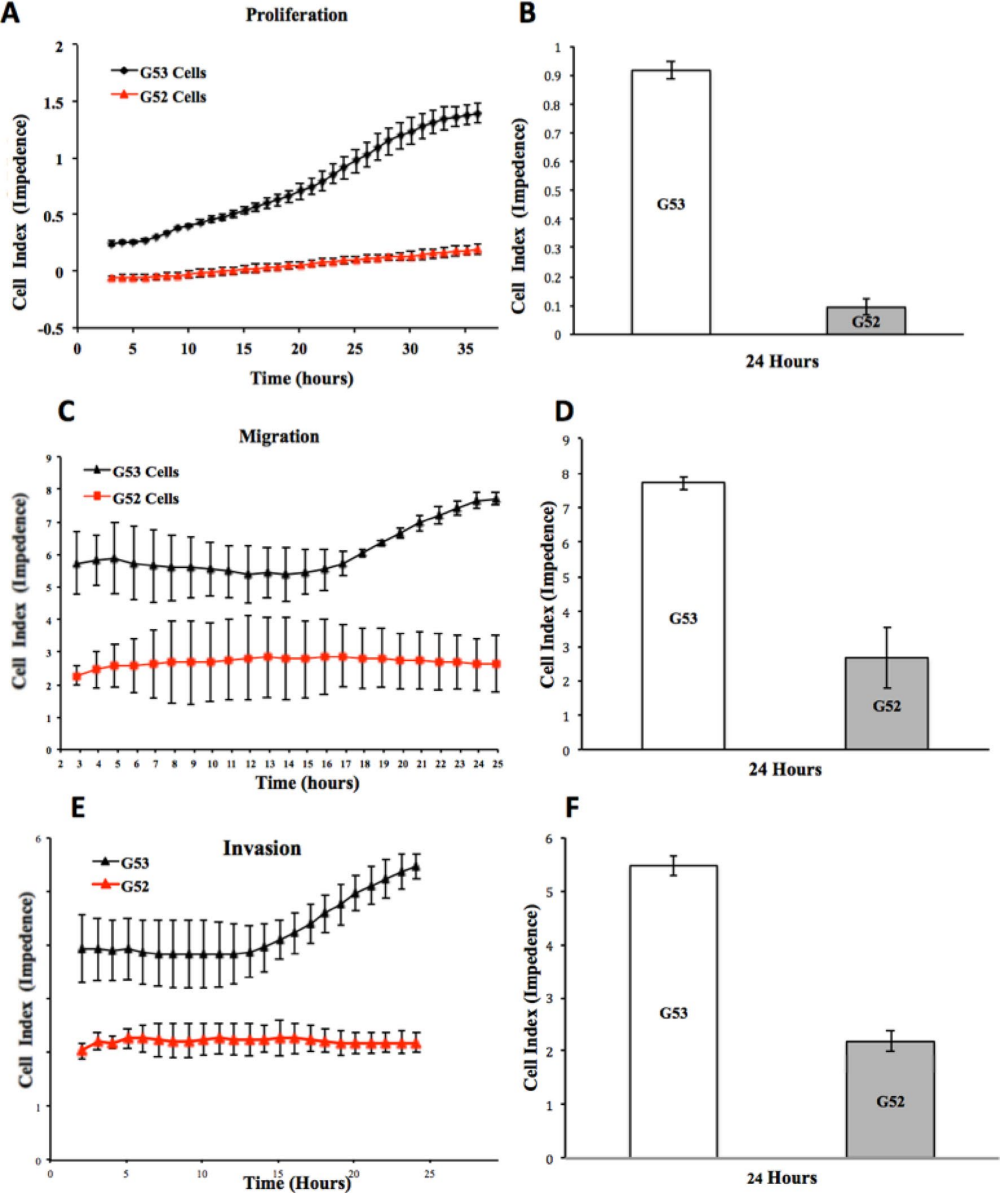
p53 mutation promotes cell proliferation, migration and invasion in multifocal primary glioblastoma cell lines in vitro. **a** Real-time xCelligence analysis of proliferation (represented by cell index) of G52 (red) and G53 (black). **b** 24 h timepoint analysis of proliferation (represented by cell index) levels between G53 and G52. Error bars represent standard deviations. *p* < 0.1; *p < 0.02. **c** Real-time xCelligence analysis of migration (represented by cell index) of G53 and G52. **d** 24 h timepoint analysis of migration (represented by cell index) levels between G53 (left frontal lesion) and G52 (left thalamic lesion). Error bars represent standard deviations. *p* < 0.1; *p* < 0.8. **e** Real-time xCelligence analysis of invasion (represented by cell index) of G53 (black) and G52 (red). **f** 24 h timepoint analysis of invasion (represented by cell index) levels between G53 (left frontal lesion) and G52 (left thalamic lesion). Error bars represent standard deviations. *p* < 0.2; *p* < 0.1

To investigate the potential functional role of the p53 mutation in G53, cell proliferation, migration and invasion were monitored in real-time using xCELLigence technology for each tumour cell line. The rate of cell proliferation was increased in the G53 cells (p53 mutant) when compared with G52 cells (p53 wt) for the same time period (Fig. 3a). At the single time-point of 24 h, the G53 cell index (CI) count was 9 × greater than G52 (Fig. 3b). The migratory properties of the tumour cells were investigated using uncoated CIM-16 xCELLigence plates. The rate of migration through the uncoated membrane was 3 × greater in G53 cells. The migratory rate of G53 continually increased after the 13 h timepoint compared to the G52 cells that maintained a lower and constant rate of migration throughout the same period (Fig. 3c). At the single timepoint of 24 h, the G53 CI count for migration was greater than G52 (Fig. 3d). The invasive properties of the cell lines were investigated using CIM-16 xCelligence plates coated with Matrigel®. The rate of cell invasion through Matrigel was increased by 2.5 x in G53 cells compared with G52 (Fig. 3e). The invasiveness of G53 cells increased sharply after 13 h. At the single timepoint of 24 h, the G53 cell index count for invasion was greater than G52 (Fig. 3f). As expected, these results indicate that the p53 mutant cell line represents a more aggressive tumour that promotes proliferation, migration and invasion of GBM cells in vitro.

### In vitro expression of wild-type and mutant p53: effect on colony formation

**Fig. 4:**
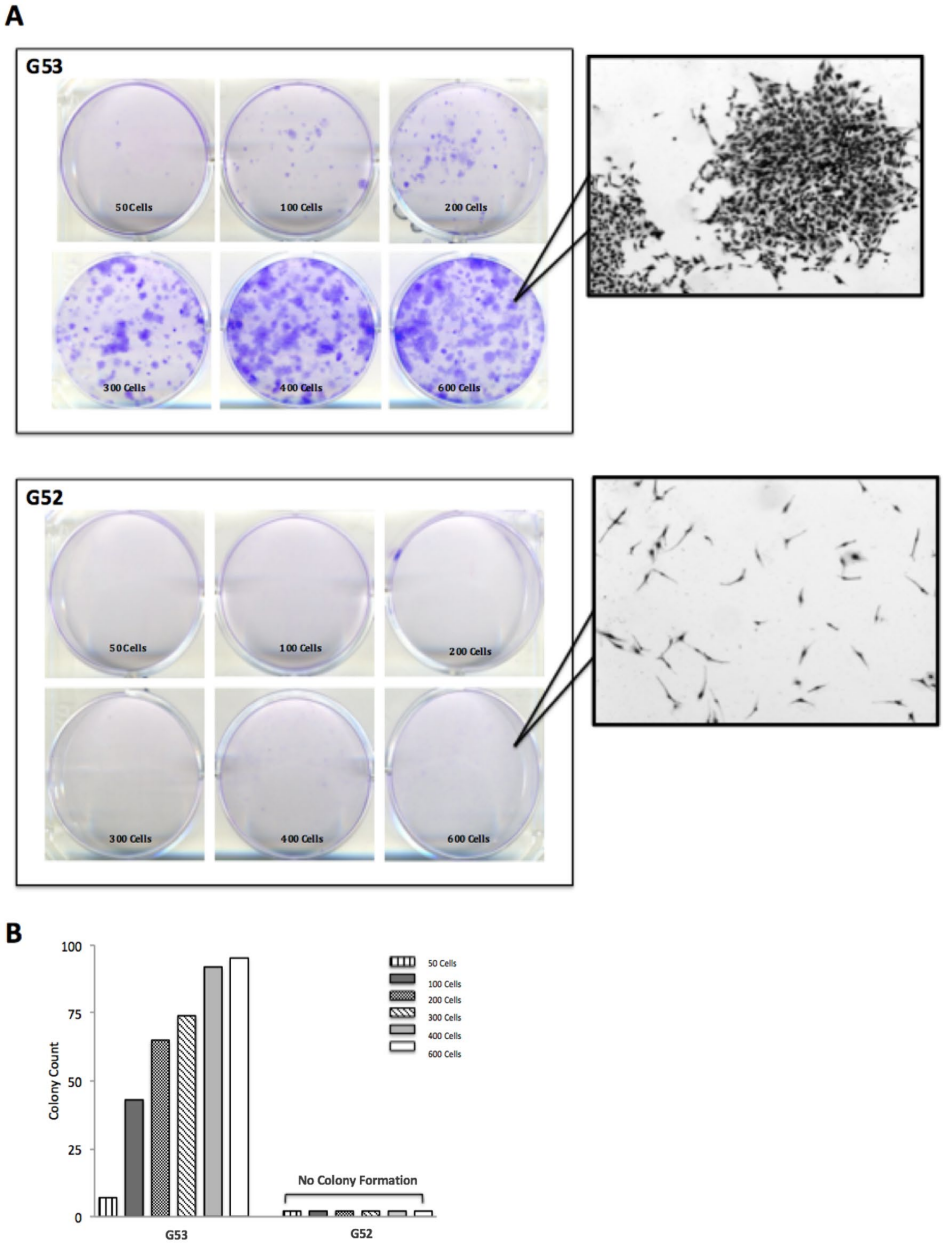
Colony formation escalation in a p53 mutant cell line. **a** G53 colony formation (crystal violet stain) increases across all cell seeding densities (50 to 600 cells) over 14 days compared to G52 (inset magnification × 20). **b** Colonies were quantified using a stereomicroscope and colony counting pen. G53 colony counts consistently increase over time with increasing cell density, whereas G52 demonstrates no change in formation

A colony formation assay was used to determine the potential and differences of both the wt and mutant p53 tumours (Fig. 4). There are clear differences between the two tumour populations with G53 forming significantly more colonies across all cell densities. Figure 4a demonstrates consistent increases in G53 colony formation (crystal violet stain) as cell density increases while G52 colonies cannot be visualised in any of the plates. These differences are quantified in Fig. 4b. These results suggest G52 maintained p53 wt tumour suppressor function whereas G53 p53 mutant supported cellular growth.

### Ki-67 expression in G52 and G53 tumour tissue

**Fig. 5:**
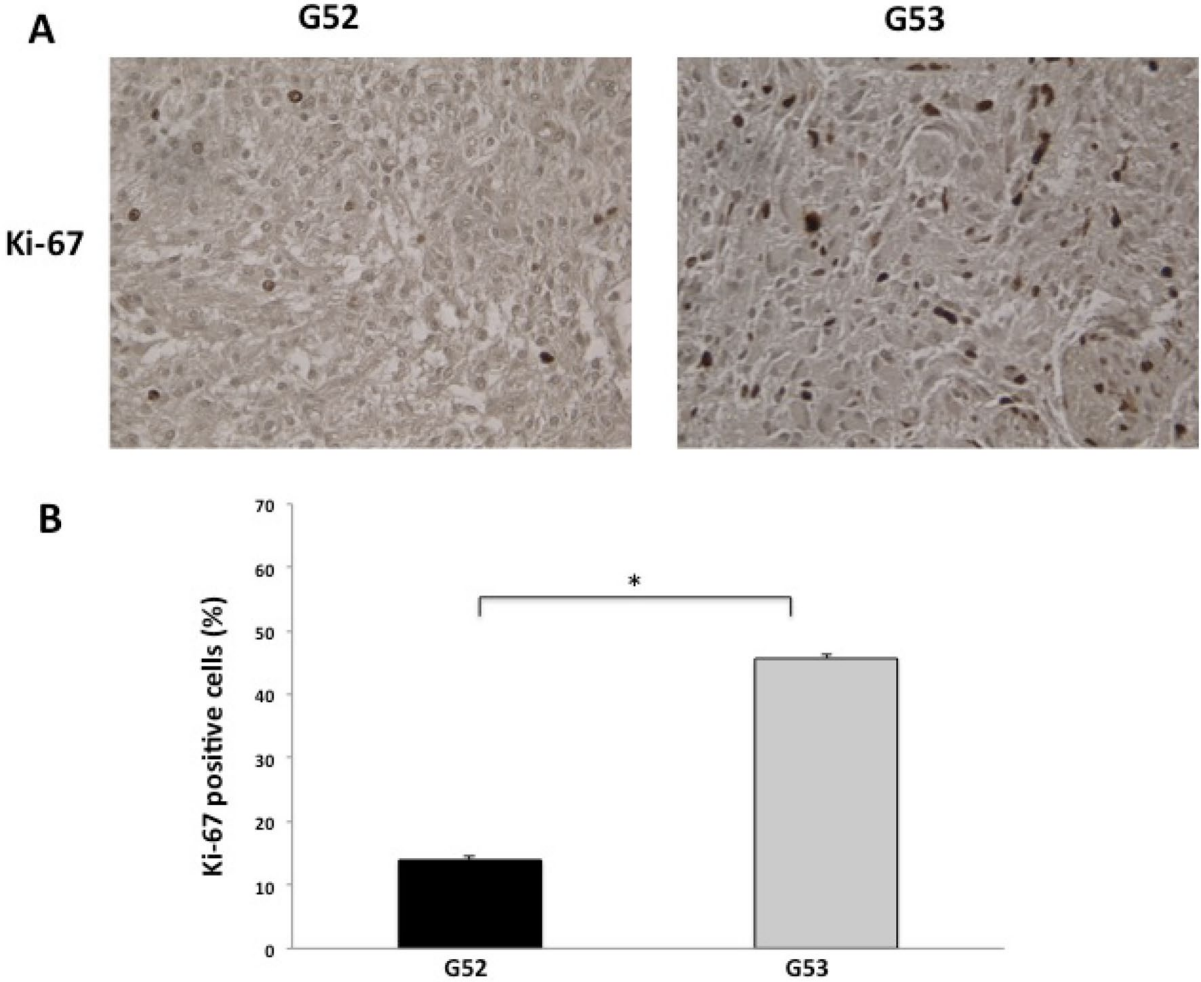
Higher cell proliferation in p53 mutant. **a** Labelling index for Ki-67 in G52 and G53 (×40 magnification). **b** LI was measured as the percentage of positive cells per 1000 cells. *p < 0.05

The labelling index (LI) for Ki-67 was calculated as the percentage of positive cells per 1000 cells (Fig. 5). The percent of Ki-67 significantly increased in G53 (*p* < 0.05; Fig. 5b), suggesting that the p53 mutation was associated with the Ki-67 LI indicating positive proliferation.

## Discussion

GBM is a molecularly complex disease resulting in extensive diagnostic and therapeutic challenges. Consequently, the standard of care has remained unchanged since the Stupp protocol was introduced in 2005, consisting of maximal safe surgical resection followed by radiotherapy plus concomitant and adjuvant temozolomide [1]. Treatment is further complicated when mGBM cases arise where the prognosis declines to 6–8 months and no standard of care exists. MC tumours are defined as lesions in different compartments of the brain with no gross or microscopic anatomical linkages. MF tumours may disseminate through established CNS routes including cerebrospinal fluid, white matter or through local invasion. However, GBMs are highly invasive and visible anatomical discontinuity does not necessarily insinuate genomic isolation between tumours. Microscopic pathways may exist that cannot be detected even through the most advanced imaging technology. Although there was no apparent FLAIR pattern of dissemination in this patient, similar genomic profiles suggest that the tumours shared a common origin, findings consistent with previous case reports [7, 17]. Moreover, GBMs are known to be a clonal disease originating from normal neural stem cells and MF cases are assumed to follow this trend through migration and mutation events [18–20].

A more intensively studied topic relative to mGBM is the *TP53* tumour suppressor gene, which confers pivotal protective functions against cancer through the regulation of cell proliferation and death. However, it is also the most commonly mutated gene found in nearly half of all human cancers. In glioma patients, *TP53* mutations have been found to have a direct negative impact on overall survival [21]. Compromises to gene functionality can occur either through attenuation via missense mutations or by allelic deletion with the former rendering a more aggressive and metastatic cancer. The majority of p53 mutants (75%) are a result of missense mutations found within the DNA-binding domain [22]. Here, they may either alter the structure of the domain (conformational mutant) or hinder contact between p53 mutants and DNA (contact mutant). Of these 75%, six frequently occur within “hotspot” codons, namely R175, G245, R248, R249, R273 and R282, which are responsible for 30% of the missense mutations [22]. These mutations result in an accumulation of highly stabilised mutant proteins within the nucleus. R273H (R270H in mice) is one of the more common and least stable of the hotspot mutations and was found to be unique in G53 [23, 24]. It is not thought to undergo conformational changes, but rather maintains the wt structure while affecting surface proteins essential for DNA binding (contact mutant) [22–24].

We anticipated distinct mutational profiles that could explain the sudden appearance and rapid development of G53. However, multiplatform profiling revealed that the positive biomarkers as well as the mutated driver genes were similar in both tumours with the only significant difference being a p53 R273H mutation in G53. Although the exact pathogenic mechanisms of MF tumours remain unknown, we hypothesise that tumour cells migrated from the left thalamus to the left temporal region despite being well-separated lesions displaying no apparent pattern of dissemination. A previous study has suggested that mGBMs are of monoclonal origin and identified potential founder events involving loss of one copy of chromosome 10 with *PTEN*, *EGFR* and *TERT* promoter mutations followed by further alterations, including *TP53* mutations [25]. The present study identified *PTEN* and *EGFR* mutations in both tumours with loss of one copy of chromosome 10 and a *TP53* mutation in G53. Due to the heterogeneic nature of GBM, we propose the possible mechanisms for which *TP53* mutated: (i) in a clonal event during a later stage of G52 tumour development before branching off to form G53 or (ii) parallel genetic evolution of a G52 tumour cell that accumulated this aberration during tumorigenesis of G53. The former is the most likely event as p53 mutant cancer cells have an increased ability to migrate to distant sites and metastasise [11]. Either scenario agrees with previous findings that suggest *TP53* is not a founder event but rather occurs during the later stages in primary GBM and early stages of secondary GBM tumour development [25, 26]. When p53 mutations do occur, they result not only in impaired tumour suppressor function, but mounting evidence has demonstrated that certain missense mutations give rise to gain-of-function (GOF) phenotypes.

Numerous studies have demonstrated the contributions of p53 GOF phenotypes to malignancy through mechanisms involving proliferation, migration, invasion, metastasis, drug resistance, colony formation, genomic instability and cancer cell survival [11, 12, 27, 28]. In vivo studies have repeatedly displayed an increase in incidence of metastatic neoplasms following the introduction of p53 R270H [24, 29–31]. We investigated both tumours in vitro to determine if the G53 p53 R273H mutation adopted GOF characteristics and contributed to its aggressive development. The assays demonstrated pronounced increases in proliferation, migration and invasion of G53 compared to G52, strongly indicating that GOF phenotypes were acquired. These results were further validated through a marked increase in colony formation of G53, suggesting the p53 mutation supports cellular growth, results coinciding with previous studies [32, 33]. Because p53 mutations inhibit DNA binding, it is believed that the initial mechanisms of GOF mutants are mediated mainly through protein–protein interactions with transcription factors such as p63 and p73, or chromatin complexes [34–37]. An alternative mechanism proposes that p53 mutants act as transcription factors with the ability to influence promoters in order to activate target genes [11]. However, the effects of p53 mutants cannot be accurately predicted and the aforementioned mechanisms are likely a combination of differing pathways. The extensive literature combined with our in vitro assays provide likely rationale that *TP53* was mutated during the later development of G52. The p53 mutant subpopulation then migrated from the left thalamus to the left temporal region where metastasis of G53 was established. The mutation provided the tumour with GOF characteristics resulting in a more aggressive tumour with greater oncogenic potential than G52. This particular case emphasises the need for genetic investigation to understand the precise disease progression of mGBM in order to create highly targeted therapies.

## Conclusion

As the most lethal brain cancer, GBM is characterised by severe genetic instability and a diffusely infiltrative nature. In this MF GBM patient, the secondary tumour acquired a p53 R273H missense mutation, thus establishing a mutational signature with evolutionary advantageous GOF phenotypes. These phenotypes likely contributed to the invasive progression and malignancy of the G53 tumour. The apparent differences in development between the tumours highlights the importance of genomic profiling, particularly in mGBM cases. Understanding each tumour individually provides further insight into the disease and may enhance personalised medicine modalities through molecularly targeted therapy.

## Electronic supplementary material

Below is the link to the electronic supplementary material.

Electronic supplementary material 1 (DOCX 62 kb)
