# Supplementary material for: The role of TP53 gain-of-function mutation in multifocal glioblastoma
Source: J Neurooncol. 2020 Jan 30;147(1):37–47. doi: 10.1007/s11060-019-03318-5 (PMC7075848; doi:10.1007/s11060-019-03318-5)
Supplement: Supplementary file 1 — Electronic supplementary material 1 (DOCX 62 kb) [file 11060_2019_3318_MOESM1_ESM.docx]

*Supplementary Material:*

**Table 1.** Illumina TruSeq Amplicon-Cancer Hotspot Panel Results

W=wild-type, I=indeterminate, U=unclassified, M=mutated

| **Gene** | **G53** | **G52** |  | **G53** | **G52** |  | **G53** | **G52** |  | **G53** | **G52** |  | **G53** | **G52** |  | **G53** | **G52** |
| --- | --- | --- | --- | --- | --- | --- | --- | --- | --- | --- | --- | --- | --- | --- | --- | --- | --- |
| ABI1 | W | W | BCL7A | W | W | CDK8 | W | W | DNMT3A | W | W | FGF10 | W | W | HEY1 | W | W |
| ABL1 | W | W | BCL9 | W | W | CDKN1B | W | W | DOT1L | W | W | FGF14 | W | W | HGF | W | W |
| ABL2 | W | W | BCOR | W | W | CDKN2A | W | W | EBF1 | W | W | FGF19 | W | W | HIP1 | W | W |
| ACKR3 | W | W | BCORL1 | U | U | CDKN2B | W | W | ECT2L | W | W | FGF23 | W | W | HIST1H3B | W | W |
| ACSL3 | W | W | BCR | U | U | CDKN2C | W | W | EGFR | M | M | FGF3 | W | W | HIST1H4I | W | W |
| ACSL6 | W | W | BIRC3 | I | I | CDX2 | W | W | EIF4A2 | W | W | FGF4 | W | W | HLF | W | W |
| AFF1 | U | U | BLM | U | U | CEBPA | W | W | ELF4 | W | W | FGF6 | W | W | HMGA1 | W | W |
| AFF3 | U | U | BMPR1A | W | W | CHCHD7 | W | W | ELK4 | W | W | FGFR1 | W | W | HMGA2 | I | I |
| AFF3 | U | U | BRAF | W | W | CHEK1 | W | W | ELL | W | W | FGFR1OP | U | U | HMGN2P46 | W | W |
| AFF3 | U | U | BRCA1 | W | W | CHEK2 | W | W | ELN | W | W | FGFR2 | W | W | HNF1A | U | U |
| AFF4 | W | W | BRCA2 | W | W | CHIC2 | I | I | EML4 | I | I | FGFR3 | W | W | HNF1A | U | U |
| AKAP9 | U | U | BRD3 | W | W | CHN1 | I | W | EP300 | W | W | FGFR4 | W | W | HNRNPA2B1 | W | W |
| AKT1 | W | W | BRD4 | W | W | CIC | W | W | EPHA3 | I | I | FH | W | W | HOOK3 | W | W |
| AKT2 | W | W | BRIP1 | W | W | CIITA | W | W | EPHA5 | I | I | FHIT | W | W | HOXA11 | W | W |
| AKT3 | I | I | BTG1 | W | W | CLP1 | W | W | EPHB1 | W | W | FIP1L1 | W | W | HOXA13 | W | W |
| ALDH2 | W | W | BTK | I | I | CLTC | W | W | EPS15 | I | I | FLCN | W | W | HOXA9 | W | W |
| ALK | W | W | BUB1B | W | W | CLTCL1 | U | U | ERBB2 | W | W | FLI1 | W | W | HOXC11 | W | W |
| AMER1 | W | W | c-KIT | W | W | cMET | W | W | ERBB3 | W | W | FLT1 | W | W | HOXC13 | W | W |
| AR | W | W | C11orf30 | W | W | CNBP | W | W | ERBB4 | I | I | FLT3 | W | W | HOXD11 | W | W |
| APC | W | W | C15orf65 | W | W | CNOT3 | W | W | ERC1 | W | W | FLT4 | W | W | HOXD13 | W | W |
| ARAF | W | W | C2orf44 | W | W | CNTRL | U | U | ERCC1 | W | W | FNBP1 | W | W | HRAS | W | W |
| ARFRP1 | W | W | CACNA1D | W | W | COL1A1 | W | W | ERCC2 | W | W | FOXA1 | W | W | HSP90AA1 | W | W |
| ARHGAP26 | W | W | CALR | W | W | COPB1 | W | W | ERCC3 | W | W | FOXL2 | W | W | HSP90AB1 | W | W |
| ARHGEF12 | W | W | CAMTA1 | I | W | COX6C | W | W | ERCC4 | W | W | FOXO1 | W | W | IDH1 | W | W |
| ARID1A | W | W | CANT1 | W | W | CREB1 | I | I | ERCC5 | U | U | FOXO3 | W | W | IDH2 | W | W |
| ARID2 | I | I | CARD11 | W | W | CREB3L1 | U | U | ERCC5 | U | U | FOXO4 | W | W | IGF1R | W | W |
| ARNT | I | I | CARS | W | W | CREB3L2 | U | U | ERG | W | W | FOXP1 | W | W | IKBKE | W | W |
| ASPSCR1 | W | W | CASC5 | I | I | CREBBP | W | W | ESR1 | U | U | FSTL3 | W | W | IKZF1 | W | W |
| ASXL1 | U | U | CASP8 | W | W | CRKL | W | W | ETV1 | W | W | FUBP1 | W | W | IL2 | I | I |
| ATF1 | W | W | CBFA2T3 | W | W | CRLF2 | W | W | ETV4 | W | W | FUS | W | W | IL21R | W | W |
| ATIC | W | W | CBFB | W | W | CRTC1 | W | W | ETV5 | W | W | GAS7 | W | W | IL6ST | W | W |
| ATM | W | W | CBL | W | W | CRTC3 | W | W | ETV6 | W | W | GATA1 | W | W | IL7R | W | W |
| ATP1A1 | W | W | CBLB | I | I | CSF1R | W | W | EWSR1 | W | W | GATA2 | W | W | INHBA | W | W |
| ATP2B3 | W | W | CBLC | W | W | CSF3R | W | W | EXT1 | W | W | GATA3 | W | W | IRF4 | W | W |
| ATR | W | W | CCDC6 | W | W | CTCF | W | W | EXT2 | W | W | GID4 | W | W | IRS2 | W | W |
| ATRX | I | I | CCNB1IP1 | W | W | CTLA4 | W | W | EZH2 | W | W | GMPS | W | W | ITK | W | W |
| AURKA | W | W | CCND1 | W | W | CTNNA1 | W | W | EZR | W | W | GNA11 | W | W | JAK1 | W | W |
| AURKB | W | W | CCND2 | W | W | CTNNB1 | W | W | FAM46C | W | W | GNA13 | W | W | JAK2 | I | I |
| AXIN1 | W | W | CCND3 | W | W | CYLD | W | W | FANCA | W | W | GNAQ | W | W | JAK3 | W | W |
| AXL | U | U | CCNE1 | W | W | CYP2D6 | W | W | FANCC | W | W | GNAS | W | W | JAZF1 | W | W |
| BAP1 | W | W | CD274 | W | W | DAXX | W | W | FANCD2 | W | W | GOLGA5 | W | W | JUN | W | W |
| BARD1 | W | W | CD74 | W | W | DDB2 | W | W | FANCE | W | W | GOPC | I | I | KAT6A | W | W |
| BCL10 | W | W | CD79A | W | W | DDIT3 | W | W | FANCF | W | W | GPC3 | W | W | KAT6B | U | U |
| BCL11A | W | W | CD79B | W | W | DDR2 | W | W | FANCG | W | W | GPHN | I | I | KCNJ5 | W | W |
| BCL11B | W | W | CDC73 | I | I | DDX10 | W | W | FANCL | I | I | GPR124 | W | W | KDM5A | W | W |
| BCL2 | W | W | CDH1 | W | W | DDX5 | W | W | FAS | W | W | GRIN2A | W | W | KDM5C | W | W |
| BCL2L11 | W | W | CDH11 | W | W | DDX6 | W | W | FBXO11 | W | W | GSK3B | I | I | KDM6A | I | I |
| BCL2L2 | U | U | CDK12 | W | W | DEK | W | W | FBXW7 | I | I | H3F3A | W | W | KDR | W | W |
| BCL3 | W | W | CDK4 | W | W | DICER1 | W | W | FCRL4 | I | W | H3F3B | W | W | KDSR | W | W |
| BCL6 | W | W | CDK6 | W | W | DNM2 | W | W | FEV | W | W | HERPUD1 | W | W | KEAP1 | W | W |

Table 1 continued:

| **Gene** | **G53** | **Gel G52** |  | **G53** | **G52** |  | **G53** | **G52** |  | **G53** | **G52** |  | **G53** | **G52** |  | **G53** | **G52** |
| --- | --- | --- | --- | --- | --- | --- | --- | --- | --- | --- | --- | --- | --- | --- | --- | --- | --- |
| KIF5B | W | W | MNX1 | W | W | NUTM2B | I | I | PSIP1 | I | I | SH2B3 | W | W | TFRC | W | W |
| KLF4 | W | W | MPL | W | W | OLIG2 | W | W | PTCH1 | W | W | SH3GL1 | W | W | TGFBR2 | W | W |
| KLHL6 | W | W | MRE11A | W | W | OMD | W | W | **PTEN** | **M** | **M** | SLC34A2 | W | W | THRAP3 | W | W |
| KLK2 | W | W | MSH2 | W | W | P2RY8 | W | W | PTPN11 | W | W | SLC45A3 | W | W | TLX1 | W | W |
| KMT2A | U | U | MSH6 | W | W | PAFAH1B2 | W | W | PTPRC | I | I | SMAD2 | W | W | TLX3 | W | W |
| KMT2C | W | W | MSI2 | W | W | PAK3 | I | I | RABEP1 | W | W | SMAD4 | W | W | TMPRSS2 | U | U |
| KMT2D | W | W | MSN | W | W | PALB2 | W | W | RAC1 | W | W | SMARCA4 | W | W | TNFAIP3 | W | W |
| KRAS | W | W | MTCP1 | W | W | PATZ1 | W | W | RAD21 | W | W | SMARCB1 | W | W | TNFRSF14 | W | W |
| KTN1 | I | I | MTOR | W | W | PAX3 | W | W | RAD50 | W | W | SMARCE1 | I | I | TNFRSF17 | W | W |
| LASP1 | W | W | MUC1 | W | W | PAX5 | W | W | RAD51 | W | W | SMO | W | W | TOP1 | W | W |
| LCK | W | W | MUTYH | W | W | PAX7 | W | W | RAD51B | W | W | SNX29 | W | W | TP53 | M | W |
| LCP1 | W | W | MYB | W | W | PAX8 | W | W | RAF1 | W | W | SOCS1 | W | W | TPM3 | W | W |
| LGR5 | W | W | MYC | W | W | PBRM1 | W | W | RALGDS | W | W | SOX10 | W | W | TPM4 | W | W |
| LHFP | W | W | MYCL | W | W | PBX1 | I | I | RANBP17 | I | I | SOX2 | W | W | TPR | W | W |
| LIFR | W | W | MYCN | W | W | PCM1 | I | I | RAP1GDS1 | W | W | SPECC1 | W | W | TRAF7 | W | W |
| LMO1 | W | W | MYD88 | W | W | PCSK7 | W | W | RARA | W | W | SPEN | W | W | TRIM26 | W | W |
| LMO2 | W | W | MYH11 | W | W | PDCD1 | W | W | RB1 | I | I | SPOP | W | W | TRIM27 | W | W |
| LPP | W | W | MYH9 | W | W | PDCD1LG2 | U | U | RBM15 | W | W | SRC | W | W | TRIM33 | I | I |
| LRIG3 | W | W | NACA | W | W | PDE4DIP | W | W | RECQL4 | U | U | SRGAP3 | W | W | TRIP11 | U | U |
| LRP1B | I | W | NBN | W | W | PDGFB | W | W | REL | W | W | SRSF2 | W | W | TRRAP | W | W |
| LYL1 | U | U | NCKIPSD | W | W | PDGFRA | W | W | RET | W | W | SRSF3 | W | W | TSC1 | W | W |
| MAF | W | W | NCOA1 | W | W | PDGFRB | W | W | RHOH | W | W | SS18 | W | W | TSC2 | W | W |
| MAFB | W | W | NCOA2 | U | U | PDK1 | W | W | RICTOR | I | I | SS18L1 | W | W | TSHR | I | I |
| MALT1 | W | W | NCOA4 | W | W | PER1 | W | W | RMI2 | W | W | SSX1 | W | W | TTL | W | W |
| MAML2 | U | U | NDRG1 | W | W | PHF6 | I | I | RNF213 | U | U | STAG2 | I | I | U2AF1 | W | W |
| MAP2K4 | I | W | NF1 | I | I | PHOX2B | W | W | RNF213 | U | U | STAT3 | W | W | UBR5 | W | W |
| MAP3K1 | M | M | NF2 | W | W | PICALM | W | W | RNF43 | W | W | STAT4 | W | W | USP6 | U | U |
| MAX | W | W | NFE2L2 | W | W | PIK3CA | I | I | ROS1 | W | W | STAT5B | W | W | USP6 | U | U |
| MCL1 | W | W | NFIB | W | W | PIK3CG | W | W | RPL10 | W | W | STIL | W | W | VEGFA | W | W |
| MDM2 | W | W | NFKB2 | W | W | PIK3R1 | W | W | RPL22 | W | W | STK11 | W | W | VEGFB | W | W |
| MDM4 | W | W | NFKBIA | W | W | PIK3R2 | W | W | RPL5 | W | W | SUFU | W | W | VHL | W | W |
| MDS2 | W | W | NIN | W | W | PIM1 | W | W | RPN1 | W | W | SUZ12 | W | W | VTI1A | W | W |
| MECOM | W | W | NKX2-1 | W | W | PLAG1 | W | W | RPTOR | W | W | SYK | W | W | WAS | W | W |
| MED12 | W | W | NONO | W | W | PML | W | W | RUNX1 | W | W | TAF15 | I | I | WHSC1 | W | W |
| MEF2B | W | W | NOTCH1 | W | W | PMS1 | W | W | RUNx1T1 | W | W | TAL1 | U | U | WHSC1L1 | W | W |
| MEK1 | W | W | NOTCH2 | U | U | PMS2 | W | W | SBDS | W | W | TAL2 | W | W | WIF1 | W | W |
| MEK2 | W | W | NOTCH2 | U | U | POLE | W | W | SDC4 | W | W | TBL1XR1 | W | W | WISP3 | W | W |
| MEN1 | W | W | NPM1 | W | W | POT1 | I | I | SDHAF2 | W | W | TCEA1 | W | W | WRN | I | I |
| MITF | W | W | NR4A3 | W | W | POU2AF1 | W | W | SDHB | W | W | TCF12 | W | W | WT1 | W | W |
| MKL1 | W | W | NRAS | W | W | POU5F1 | W | W | SDHC | W | W | TCF3 | W | W | WWTR1 | W | W |
| MLF1 | W | W | NSD1 | W | W | PPARG | W | W | SDHD | W | W | TCF7L2 | W | W | XPA | W | W |
| MLH1 | W | W | NT5C2 | W | W | PPP2R1A | W | W | SEP5 | W | W | TCL1A | W | W | XPC | U | U |
| MLLT1 | W | W | NTRK1 | M | M | PRCC | W | W | SEP6 | W | W | TERT | W | W | XPO1 | W | W |
| MLLT10 | I | I | NTRK2 | W | W | PRDM1 | W | W | SEP9 | W | W | TET1 | W | W | YWHAE | W | W |
| MLLT11 | W | W | NUMA1 | W | W | PRDM16 | W | W | SET | W | W | TET2 | W | W | ZBTB16 | W | W |
| MLLT3 | W | W | NUP214 | W | W | PRF1 | W | W | SETBP1 | W | W | TFE3 | W | W | ZMYM2 | W | W |
| MLLT4 | W | W | NUP93 | W | W | PRKAR1A | W | W | SETD2 | W | W | TFEB | W | W | ZNF217 | U | U |
| MLLT6 | U | U | NUP98 | W | W | PRKDC | W | W | SF3B1 | W | W | TFG | W | W | ZNF331 | U | U |
| MN1 | U | U | NUTM1 | W | W | PRRX1 | W | W | SFPQ | W | W | TFPT | W | W | ZNF384 | U | U |
